# Human Bone Marrow Mesenchymal Stromal Cell-Derived Extracellular Vesicles Promote Proliferation of Degenerated Nucleus Pulposus Cells and the Synthesis of Extracellular Matrix Through the SOX4/Wnt/β-Catenin Axis

**DOI:** 10.3389/fphys.2021.723220

**Published:** 2021-10-28

**Authors:** Haoyu Wang, Fei Li, Wenrui Ban, Jing Zhang, Guiqi Zhang

**Affiliations:** ^1^Department of Orthopedics, The Second Affiliated Hospital, Xi’an Jiaotong University, Xi’an, China; ^2^Department of Spinal Surgery, Dalian Municipal Central Hospital, Dalian, China

**Keywords:** intervertebral disk degeneration, extracellular vesicles, degenerated nucleus pulposus cells, human bone marrow mesenchymal stromal cells, miR-129-5p, SRY-box transcription factor 4, Wnt/β-catenin, extracellular matrix

## Abstract

**Objective:** Intervertebral disk degeneration (IDD) is a major cause of pain in the back, neck, and radiculus. Mesenchymal stem cells (MSCs)-derived extracellular vesicles (EVs) are therapeutic in musculoskeletal degenerative diseases such as IDD. This study explored the effect and functional mechanism of human bone MSCs (hBMSCs)-derived EVs in proliferation and apoptosis of degenerated nucleus pulposus cells (DNPCs) and extracellular matrix (ECM) synthesis.

**Methods:** Extracellular vesicles were isolated from hBMSCs and identified. DNPCs were induced by TNF-α. EVs were incubated with DNPCs for 24h. Internalization of EVs by DNPCs, DNPCs proliferation, apoptosis, and expressions of ECM synthetic genes, degrading genes and miR-129-5p were assessed. Downstream target genes of miR-129-5p were predicted. Target relation between miR-129-5p and SRY-box transcription factor 4 (SOX4) was verified. DNPCs proliferation, apoptosis, and ECM synthesis were measured after treatment with EVs and miR-129-5p inhibitor or SOX4 overexpression. Expressions of SOX4 and Wnt/β-catenin pathway-related proteins were determined.

**Results:** hBMSC-EVs promoted DNPCs proliferation, inhibited apoptosis, increased expressions of ECM synthetic genes, and reduced expressions of ECM degrading genes. hBMSC-EVs carried miR-129-5p into DNPCs. Silencing miR-129-5p in EVs partially inverted the effect of EVs on DNPCs proliferation and ECM synthesis. miR-129-5p targeted SOX4. SOX4 overexpression annulled the effect of EVs on DNPCs proliferation and ECM synthesis. Expressions of Wnt1 and β-catenin were decreased in EVs-treated DNPCs, while silencing miR-129-5p in EVs promoted expressions of Wnt1 and β-catenin.

**Conclusion:** hBMSC-EVs promoted DNPCs proliferation and ECM synthesis by carrying miR-129-5p into DNPCs to target SOX4 and deactivating the Wnt/β-catenin axis.

## Introduction

Intervertebral disk degeneration (IDD) is a common chronic disease leading to a series of clinical symptoms including cervical spondylitis and intervertebral disk herniation (Chan and Gantenbein-Ritter, 2012; Kos et al., 2019). A recent study has revealed that IDD will be initiated under various physiological and pathological conditions and can be influenced by a range of factors including genetics, cell senescence, mechanical load, extracellular matrix (ECM) degradation, inflammation, and apoptosis (Stokes and Iatridis, 2004; Galbusera et al., 2014). IDD is characterized by ECM loss and apoptosis of nucleus pulposus cells (NPCs; Kadow et al., 2015). However, the exact mechanism of IDD remains unknown. It is difficult to reverse IDD progression once occurred, and there is no effective method to prevent the degenerative changes in IDD (Sakai and Schol, 2017). Traditional treatment methods of IDD such as conservative treatment and surgical intervention can only mitigate low back pain, worse yet, often cause recurrence on the same or adjacent sites and the degeneration of intervertebral disk is not eliminated (Richardson et al., 2007). Therefore, it is warranted to explore more effective therapeutic strategies for IDD.

Bone marrow mesenchymal stromal cells (BMSCs) are the main source of mesenchymal stromal cells (MSCs) which secrete a variety of cytokines and growth factors (Strioga et al., 2012). The therapeutic effect of BMSCs on the occurrence of IDD has attracted researcher’s attention recently due to their ability to suppress NPC apoptosis (Yang et al., 2010; Richardson et al., 2016). Extracellular vesicles are a group of cell-derived membranous structures which comprises exosomes and microvesicles (van Niel et al., 2018). EVs mediate intercellular communication by carrying proteins, RNA species, lipids, and other biomacromolecules from parent cells to target cells (El Andaloussi et al., 2013). BMSCs have been reported to transfer EVs to protect against tissue or organ damage (Eirin et al., 2017; Wang et al., 2018b). BMSC-EVs repress NPC apoptosis induced by inflammatory cytokines (Lu et al., 2017; Cheng et al., 2018) but the effect and mechanism of BMSC-EVs in IDD remain unclear.

microRNAs (miRNAs) are a kind of small non-coding RNAs regulating target gene expressions and have been reported to participate in IDD development (Wang et al., 2015; Lan et al., 2021). Currently, the study about BMSC-EVs affecting NPC function *via* miRNAs is in its infancy. Wen et al. (2021) have reported that BMSC-EVs promote IDD repair by transferring miR-199a. Zhu et al. (2020) have shown that exosomal miR-532-5p released from BMSCs delays IDD by targeting RASSF5. Existing studies have revealed the association of miR-129-5p with IDD and the functionality of miR-129-5p overexpression to inhibit NPC apoptosis and degrade IDD risk by modulating FADD/BMP-2 (Yang and Sun, 2019; Li et al., 2020). SRY-box transcription factor 4 (SOX4) has been documented to avert the effect of miR-499a-5p on NPC proliferation and ECM synthesis (Sun et al., 2019). The Wnt/β-catenin pathway is associated with NPC senescence and apoptosis and ECM degradation (Chen et al., 2017). However, whether BMSC-EVs regulate NPCs in IDD through the miR-129-5p/SOX4/Wnt/β-catenin axis has not been reported. This study investigated the functional mechanism of BMSC-EVs on degenerated NPCs (DNPCs) proliferation and apoptosis and ECM synthesis with the purpose to provide new reference for IDD management.

## Materials and Methods

### Culture and Identification of Human BMSCs

Human BMSCs (hBMSCs) purchased from Cyagen Biosciences (Guangzhou, China) were cultured in Dulbecco’s modified Eagle’s medium (DMEM)/F12 medium (Gibco, Grand Island, NY, United States) in an incubator at 37°C with 5% CO_2_. Once reaching 80% confluence, the immunophenotypes of hBMSCs were identified. Specifically, about 2×10^5^ hBMSCs were collected and centrifuged at 150*g* for 5min with the supernatant removed. Next, the cells were resuspended with phosphate buffer saline (PBS), and 100 μl suspension was transferred to an Eppendorf (EP) tube. Fluorescein isothiocyanate (FITC)-labeled monoclonal antibodies (CD29, CD34, CD44, and CD90; BD Bioscience, Franklin Lakes, NJ, United States) were added into cell suspension and reacted in the dark for 30–60min (with IgG as an isotype control). After three times of PBS washes, cells were suspended in 450μl PBS and detected using flow cytometry.

Next, the adipogenic and osteogenic abilities of hBMSCs were determined. For adipogenic differentiation, 2×10^4^ hBMSCs were seeded into 6-well plates and cultured in adipogenic differentiation basic culture medium A (Cyagen Biosciences) for 3days and in adipogenic differentiation basic culture medium B (Cyagen Biosciences) for 1day. The procedures were repeated three times. The hBMSCs were fixed with 4% paraformaldehyde, stained with Oil red O (Sigma-Aldrich, St. Louis, MO, United States) for 30min, and observed under an inverted microscope (Olympus, Tokyo, Japan). For osteogenic differentiation, 3×10^4^ hBMSCs were seeded into 6-well plates and cultured in osteogenic differentiation medium (Cyagen Biosciences) for 4weeks. Subsequently, hBMSCs were fixed with 4% paraformaldehyde and stained with 1% Alizarin red (Sigma-Aldrich) for 5min. Mineralized nodule formation was observed under the inverted microscope (Olympus).

### Isolation and Identification of hBMSC-EVs

The supernatant was removed when the confluence of hBMSCs reached around 80%. Subsequently, 10% EV-free fetal bovine serum (FBS; EVs in FBS were removed by centrifugation at 100,000g for 10min) was added and cultured in the CO_2_ incubator for 48h at 37°C. EVs were extracted using ultra-high-speed centrifugation (Wu et al., 2020). When the medium was changed, the supernatant was collected and centrifuged for 10min at 4°C at 500g, followed by another centrifugation for 20min at 12,000g. The supernatant was centrifuged at ultra-high speed for 2h at 100,000g using a 0.22μM filtration membrane. The precipitation was resuspended with PBS and then centrifuged at ultra-high speed for 2h, followed by resuspension with PBS and storage at −80°C. The isolated EVs were identified after the morphology of EVs was observed under a transmission electron microscope (TEM), and the size distribution of EVs was analyzed using nanoparticle tracking analysis (NTA), and the expression of CD9 and CD63 was detected on the surface of BMSC-EVs *via* Western blot. Additionally, hBMSC supernatant added with EV inhibitor GW4869 (20μg/ml conditioned medium; Sigma-Aldrich) was used as a control (GW). The identified EVs were lysed with the lysis buffer. Total protein content was determined using the bicinchoninic acid (BCA) kit (23225, Thermo Fisher Scientific, Shanghai, China).

EVs used in this study were divided into six groups: GW group (hBMSC supernatant added with GW4869), EVs group, EVs+RNase group (EVs added with RNase), EVs+RNase+SDS group (EVs added with RNase and lysis buffer SDS), EVs-NC group (EVs isolated from hBMSCs transfected with negative control (NC) of miR-129-5p inhibitor for 24h), and EVs+inhi group (EVs isolated from hBMSCs transfected with miR-129-5p inhibitor for 24h). hBMSC transfection was conducted in strict conformity with the manufacturer’s instructions. Briefly, miR-129-5p inhibitor or miR-129-5p inhibitor NC (GenePharma, Shanghai, China) was transfected into hBMSCs using Lipofectamine 2000 (Invitrogen, Carlsbad, CA, United States). Following experiments were performed after 24h.

### Treatment and Grouping of NPCs

Nucleus pulposus cells (ScienCell 4800; American Science Cell Research Laboratories, Carlsbad, CA, United States) were cultured in DMEM/F12 medium containing 10% FBS with 5% CO_2_ at 37°C. DNPCs were induced by tumor necrosis factor-α (TNF-α) according to a previous study (Chen et al., 2017). In particular, NPCs at the logarithmic growth phase were harvested and seeded into DMEM/F12 medium containing 10% FBS added with 10ng/ml TNF-α at 5×10^5^ cells/ml and cultured with 5% CO_2_ at 37°C for 24h. DNPCs were assigned into seven groups: blank group (DNPCs were added with an equal amount of PBS), DNPCs+GW group (DNPCs were treated with 50μg GW for 24h), DNPCs+EVs group (DNPCs were treated with 50μg EVs from the EVs group for 24h), DNPCs+EVs-NC group (DNPCs were treated with 50μg EVs from the EVs-NC group for 24h), DNPCs+EVs-inhi group (DNPCs were treated with 50μg EVs from the EVs-inhi group for 24h), DNPCs-oe-NC+EVs group (DNPCs were transfected with pcNDA-NC and treated with 50μg EVs from the EVs group), and DNPCs-oe-SOX4+EVs group (DNPCs were transfected with pcDNA-SOX4 and treated with 50μg EVs from the EVs group).

### Immunocytochemistry

Nucleus pulposus cells and DNPCs climbing sheets were fixed with 4% paraformaldehyde (Beyotime, Shanghai, China) for 30min, rinsed with PBS and immersed with 20% H_2_O_2_ methanol solution for 30min at room temperature. After blockade with 1:10 horse serum (Beyotime) for 20min at room temperature, the serum was removed, and cells were added with Collagen II (Col II) primary antibody (1:500, ab34712; Abcam, Cambridge, MA, United States) and incubated at 4°C overnight. Cells were then added with secondary antibody IgG (1:2,000, ab205718; Abcam) and incubated for 1h at room temperature, followed by 20-min incubation with SABC reagent (Maxim Corp, Fuzhou, China) in the 37°C constant temperature water bath. After color development using 2,4-diaminobutyric acid (DAB), cells were counterstained with hematoxylin (Beyotime) and observed under a microscope (TS100; Nikon, Kanagawa, Japan).

### Internalization of Dil-EVs Into DNPCs

Dil-labeled EVs were incubated with DNPCs for 24h. After twice rinsing with PBS, NPCs were fixed with 4% paraformaldehyde and counterstained with 4′,6-diamidino-2-phenylindole (DAPI; Beyotime). After staining, cells were observed using BX53 fluorescence microscope (×400, Olympus).

### Reverse Transcription-Quantitative Polymerase Chain Reaction (RT-qPCR)

Total RNA was extracted from cells using a TRIzol kit (Invitrogen) as per the instructions. cDNA template was synthesized from total RNA using Transcriptor First Strand cDNA Synthesis kit (TaKaRa Biotechnology, Dalian, China). TaqMan sequence and probe were obtained from Takara (Tokyo, Japan). Quantitative PCR was conducted using SYBR Green II (TaKaRa) with ABI PRISM 7900 Sequential Detection System under the reaction condition of pre-denaturation at 95°C for 10min and 40cycles of denaturation at 95°C for 10s, annealing at 60°C for 20s and extension at 72°C for 34s with U6 as an internal control. Data were analyzed using the 2^−ΔΔ*C*t^ method. Primer sequences (Sangon Biotech, Shanghai, China) are given in Table 1.

**Table 1:** Primer sequences.

Gene	Forward 5′–3′	Reverse 5′–3′
*miR-129-5p*	CAGGAAGCCCACCCCAA	AGTGCAGGGTCCGAGGTATT
*U6*	ATTGGAACGATACAGAGAAGATT	GGAACGCTTCACGAATTTC

miR, microRNA.

### Western Blot

Cells or EVs were added into radio immunoprecipitation assay lysis buffer containing protease inhibitor cocktail (Sigma-Aldrich) and mixed thoroughly, lysed on ice for 30min and centrifuged for 10min at 13,000rpm with the supernatant removed. Protein concentration was determined using the BCA kit (Pierce, Rockford, IL, United States). The proteins were separated using the prepared 10% sodium dodecyl sulfate-polyacrylamide gel electrophoresis and transferred onto the polyvinylidene fluoride (PVDF) membranes. The membranes were washed in tris-buffered saline-Tween-20 (TBST)-configured 5% skim milk and blocked on a shaking table for 1h. The membranes were then incubated with primary antibodies against Aggrecan (ab3778, 1:100), Col II (ab184993, 1:1,000), MMP3 (ab53015, 1:1,000), ADAMTS5 (ab41037, 1:250), SOX4 (ab86809, 1:100), Wnt1 (ab15251, 1:25), and β-catenin (ab32572, 1:500) at 4°C overnight. After three times of TBST washing, the membranes were incubated with horseradish peroxidase-labeled secondary antibody for 1h at room temperature. Finally, the protein bands were visualized using an enhanced chemiluminescence kit (Pierce) and detected using Image J software 1.48 (NIH, Bethesda, MD, United States) with GAPDH as an internal control.

### CCK-8 Assay

CCK-8 assay was adopted to assess cell proliferation. Cells were seeded into 96-well plates (1,500 cells/well) after transfection, and added with CCK-8 solution (Dojindo Molecular Technologies, Kumamoto, Japan) at baseline (day 0) and on day 1, 2, 3, 4 and incubated with 5% CO_2_ for 2h at 37°C. The optical density value of each well at 450nm was detected using a microplate reader (Tecan, Mannedorf, Switzerland).

### Flow Cytometry

Degenerated nucleus pulposus cells at the third passage under good growth conditions were detached with trypsin. The cell concentration was adjusted to 1×10^6^ cells/ml. After washing with PBS, 1ml cells were mixed with 100ml mouse anti-human CD24-phycoerythrin (PE) monoclonal antibody with the isotype control set. Cells were incubated for 30min at room temperature under conditions devoid of light, washed with 2ml PBS and then counted using flow cytometry. The positive rate of CD24 in DNPCs was documented based on the fluorescence intensity with the isotype control as the negative cell group.

### Bioinformatics Analysis

The downstream target genes of miR-129-5p were predicted through the StarBase,^1^ TargetScan Release 7.1,^2^ RNAInter (Score>0.9),^3^ and miRDB (Score≥90).^4^

### Dual-Luciferase Reporter Assay

The complementary sequence and mutant sequence of miR-129-5p and SOX4 were amplified and cloned onto pmiR-GLO luciferase vectors (Promega, Madison, WI, United States) to construct wild-type plasmid SOX4-WT and corresponding mutant plasmid SOX4-MUT. NPCs were seeded in 12-well plates (5×10^4^ cells/well) and cultured overnight. SOX4-WT/SOX4-MUT (Shanghai Jieneng Technology Co., Ltd., Shanghai, China) and miR-129-5p mimic or NC (GenePharma) were transfected into NPCs using Lipofectamine 2000 (Invitrogen). After 48-h transfection, NPCs were washed with PBS and lysed. Luciferase activity was detected using dual-luciferase assay detection kit (Promega).

### Statistical Analysis

SPSS 21.0 (IBM Corp., Armonk, NY, United States) and GraphPad Prism 8.01 (GraphPad Software Inc., San Diego, CA, United States) were used for data analysis and graph plotting. Normality and homogeneity of variance tests were performed first to verify that the data were consistent with normal distribution and homogeneity of variance. Data were presented as mean±standard error. Pairwise comparisons were analyzed using the independent sample *t* test, while comparisons among groups were analyzed using one-way analysis of variance (ANOVA), followed by Tukey’s multiple comparisons test. *p*<0.05 was indicative of statistical significance.

## Results

### Identification of hBMSCs and EVs

**Figure 1:**
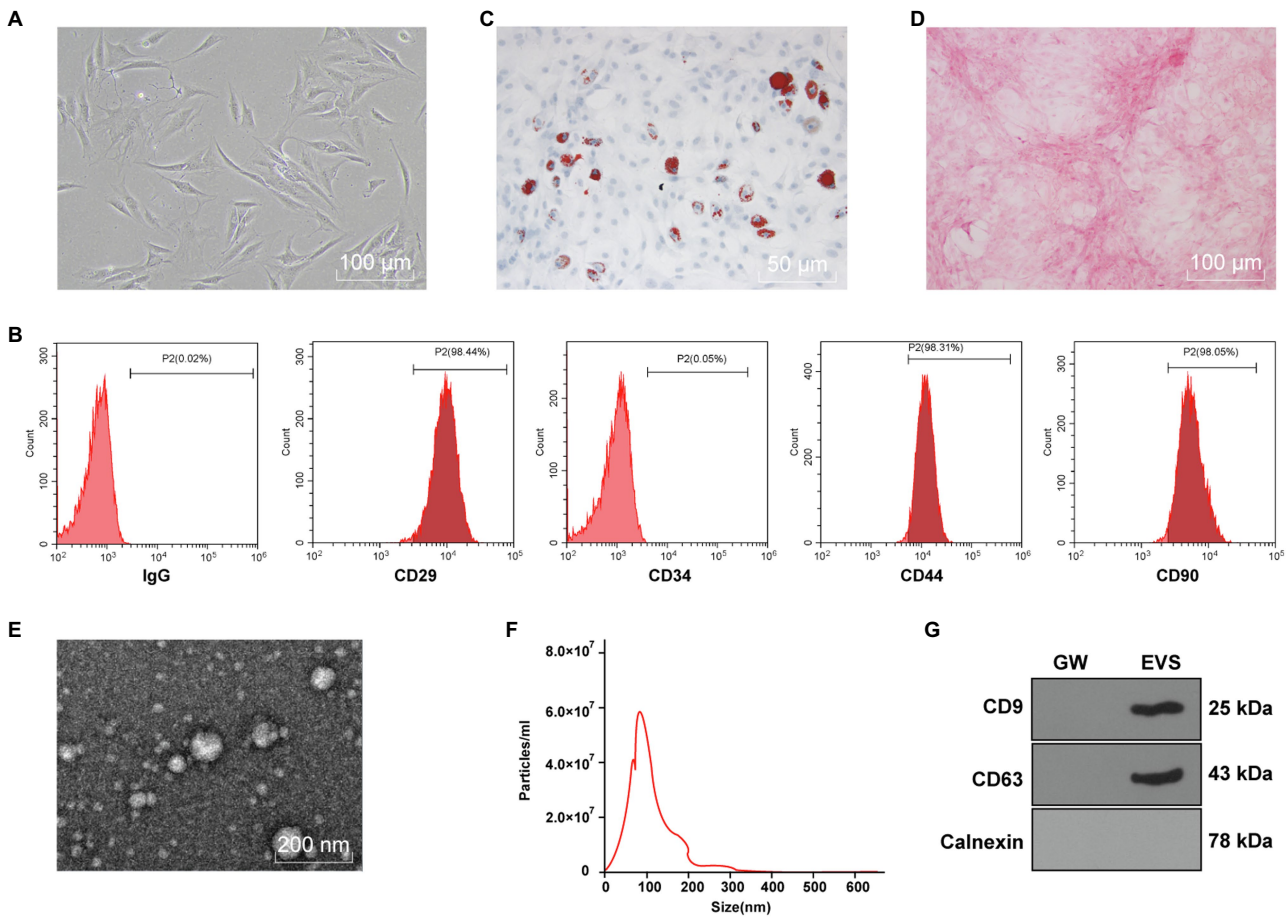
Identification of human bone marrow mesenchymal stromal cells (hBMSCs) and extracellular vesicles (EVs). The purchased hBMSCs were purified. **(A)** morphology of hBMSCs observed under a microscope; **(B)** surface antigens in hBMSCs detected by flow cytometry; **(C)** lipid differentiation observed by Oil red O staining; **(D)** osteogenic differentiation observed by Alizarin red staining; **(E)** morphology of EVs isolated from hBMSCs using ultra-high-speed centrifugation observed under transmission electron microscope (TEM); **(F)** EV size distribution analyzed by nanoparticle tracking analysis (NTA); **(G)** expressions of CD9, CD63, and Calnexin in EVs detected by Western blot. Cell experiment was repeated three times.

The purchased hBMSCs were initially purified for three generations to obtain relatively uniform and active cells, which were in spindle shapes and arranged in a whirlpool pattern (Figure 1A). Flow cytometry showed that expressions of CD29, CD44, and CD90 in hBMSCs were positive, while CD34 expression was negative (Figure 1B). Moreover, Oil red O staining after adipogenic differentiation induction of hBMSCs verified lipid deposition, which reflected the lipid differentiation ability of hBMSCs (Figure 1C). hBMSCs were stained red by Alizarin red after osteogenic differentiation induction (Figure 1D).

Subsequently, EVs were isolated from hBMSCs with ultra-high-speed centrifugation. TEM showed that EVs were in round or oval shape with non-uniform granules and membranous structure (Figure 1E). NTA was conducted to further clarify the size distribution, and the result showed that EVs were about 100nm in size with the concentration of 5.8×10^7^ particles/ml (Figure 1F). Western blot showed that CD9 and CD63 were enriched in isolated EVs, while Calnexin was not expressed (Figure 1G). These results indicated that hBMSC-EVs were successfully obtained.

### hBMSC-EVs Promoted DNPCs Proliferation and ECM Synthesis

**Figure 2:**
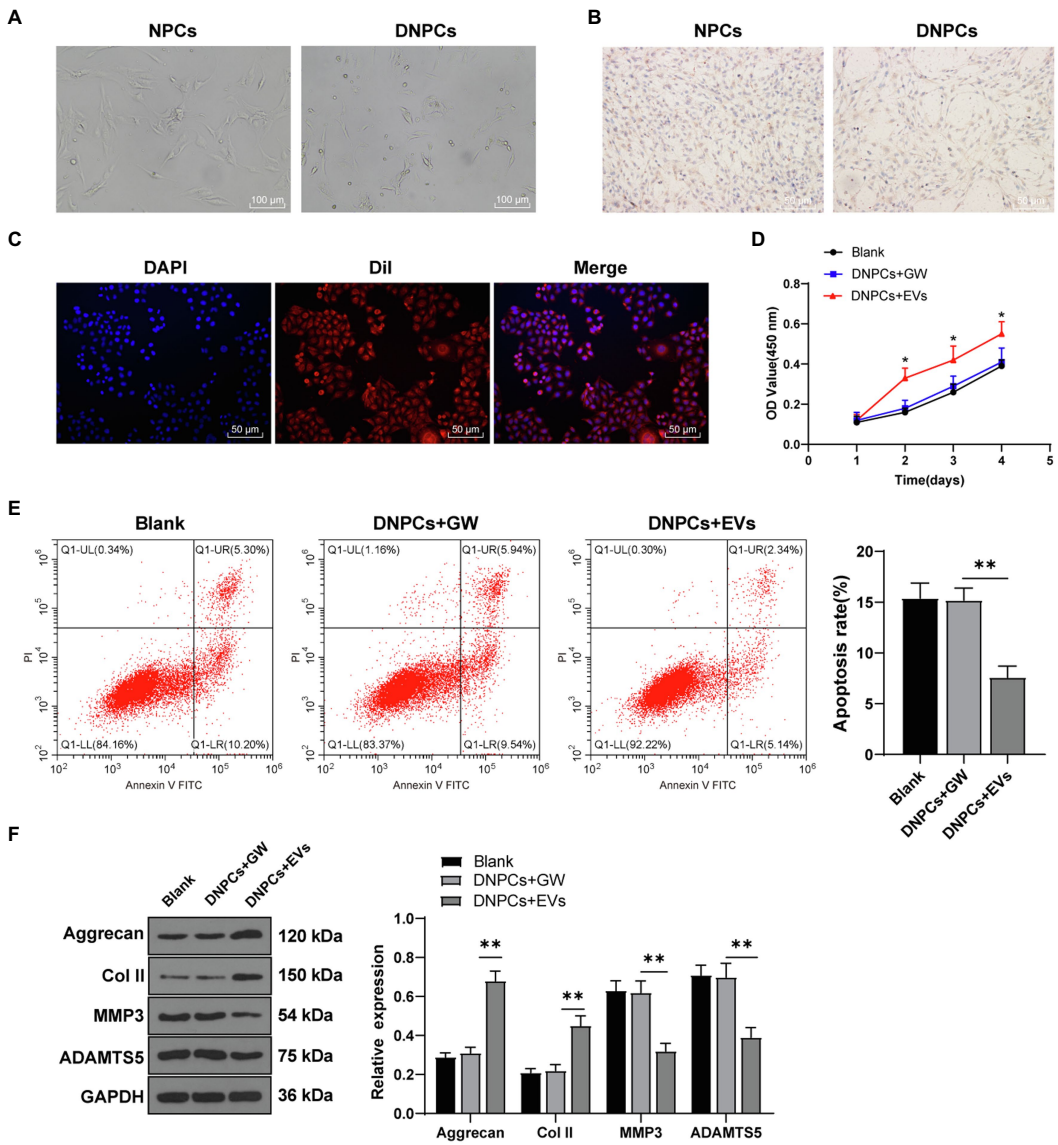
hBMSC-EVs promoted degenerated nucleus pulposus cells (DNPCs) proliferation and extracellular matrix (ECM) synthesis. DNPCs were induced by tumor necrosis factor-α (TNF-α). **(A)** morphology of NPCs and DNPCs observed under a microscope; **(B)** expression of Col II detected by immunofluorescence; **(C)** internalization of Dil-labeled EVs by DNPCs detected by immunofluorescence after incubating EVs with DNPCs for 24h; **(D)** DNPCs proliferation detected by CCK-8 assay; **(E)** DNPCs apoptosis detected by flow cytometry; **(F)** expressions of ECM synthetic genes Aggrecan and Col II and ECM degrading genes MMP3 and ADAMTS5 detected by Western blot. Cell experiment was repeated three times. Data were presented as mean±standard error. Comparisons among groups were analyzed using one-way ANOVA, followed by Tukey’s multiple comparisons test. ^*^p<0.05, ^**^p<0.01.

To study the effect of EVs on DNPCs, DNPCs were firstly obtained through TNF-α induction. NPCs observed under the microscope exhibited clear morphology of short spindle and polygonal shape, while DNPCs were in spindle and irregular shape (Figure 2A). Col II is the most abundant collagen in nucleus pulposus tissues. Immunocytochemistry showed that the cytoplasm of NPCs was stained dark tan, while the cytoplasm of DNPCs were stained light yellow, which was in consistency with the degeneration of Col II synthesis (Figure 2B). These results suggested that correct and usable DNPCs were obtained.

Next, Dil-labeled EVs were incubated with DNPCs. After 24h, immunofluorescence results demonstrated red fluorescence in DNPCs (Figure 2C), suggesting that EVs were internalized by DNPCs. Furthermore, DNPCs were treated with EVs and hBMSC supernatant added with GW4869 (GW group) for 24h. CCK-8 assay showed that relative to the blank group and GW group, the proliferative ability of DNPCs was enhanced after EV treatment (all *p*<0.05, Figure 2D). Flow cytometry showed that DNPCs apoptosis was decreased after EV treatment compared with the blank group and GW group (all *p*<0.01, Figure 2E). In addition, it has been reported that excessive ECM destruction, loss of Col II and Aggrecan in particular, contributes significantly to the occurrence and development of IDD (Sivan et al., 2014). Western blot showed that expressions of Aggrecan and Col II were increased, while expressions of MMP3 and ADAMTS5 were decreased in DNPCs after EV treatment (all *p*<0.01, Figure 2F), suggesting that EVs promoted ECM synthesis in DNPCs. Above results demonstrated that hBMSC-EVs promoted DNPCs proliferation and ECM synthesis and inhibited apoptosis.

### EVs Carried miR-129-5p Into DNPCs

**Figure 3:**
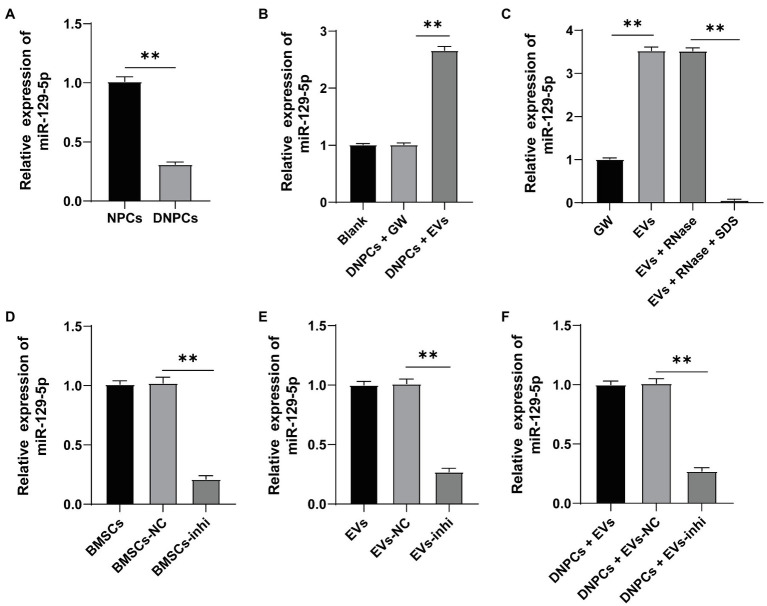
Extracellular vesicles carried miR-129-5p into DNPCs. **(A)** miR-129-5p expression in NPCs and DNPCs detected by reverse transcription-quantitative polymerase chain reaction (RT-qPCR); **(B)** miR-129-5p expression in EVs-treated DNPCs detected by RT-qPCR; **(C)** miR-129-5p expression in EVs detected by RT-qPCR; hBMSCs were transfected with miR-129-5p inhibitor and EVs were isolated, and RT-qPCR was performed to detect **(D)** miR-129-5p expression in hBMSCs; **(E)** miR-129-5p expression in EVs; **(F)** miR-129-5p expression in DNPCs. Cell experiment was repeated three times. Data were presented as mean±standard error. Data in panel **(A)** were analyzed using independent sample *t* test, while data in panels **(B–F)** were analyzed using one-way ANOVA, followed by Tukey’s multiple comparisons test. ^**^p<0.01.

miR-129-5p is associated with IDD and upregulation of miR-129-5p inhibits NPC apoptosis in IDD and reduces IDD risk by regulating FADD/BMP-2 (Yang and Sun, 2019; Li et al., 2020). RT-qPCR showed that miR-129-5p expression was decreased in DNPCs relative to that in NPCs (*p*<0.01), while miR-129-5p expression was elevated in DNPCs after EV treatment (Figures 3A,B), and miR-129-5p expression in hBMSC-EVs was higher than that in hBMSC supernatant treated with GW4869 (*p*<0.01, Figure 3C). Herein, we speculated that hBMSC-EVs affect DNPCs proliferation and apoptosis and ECM synthesis by carrying miR-129-5p. After treating EVs with RNase, miR-129-5p expression in EVs showed no significant difference, while miR-129-5p expression was considerably decreased after treatment with lysis buffer SDS and RNase (all *p*<0.01, Figure 3C), which elicited that miR-129-5p was encapsulated in EVs. Then, hBMSCs were transfected with miR-129-5p inhibitor and then EVs were isolated. miR-129-5p was downregulated in hBMSCs and EVs after transfection (*p*<0.01, Figures 3D,E). Subsequently, DNPCs were treated with EVs-inhi, and the result showed that miR-129-5p expression was reduced in the DNPCs+EVs-inhi group relative to that in the DNPCs+EVs-NC group (all *p*<0.01, Figure 3F). These results elucidated that hBMSC-EVs carried miR-129-5p into DNPCs.

### Silencing miR-129-5p Inverted the Promoting Effect of EVs on DNPCs Proliferation and ECM Synthesis

**Figure 4:**
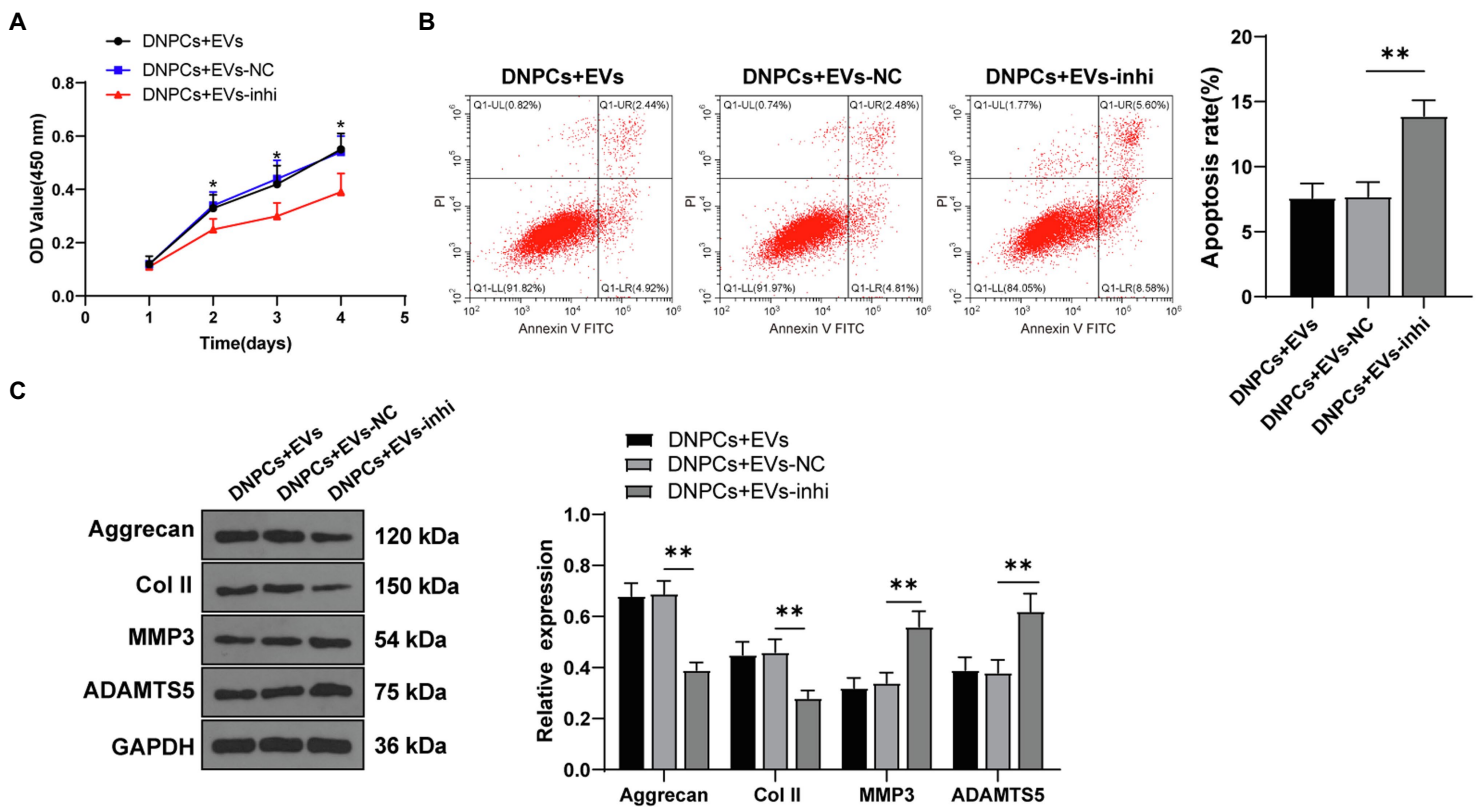
Silencing miR-129-5p inverted the promoting effect of EVs on DNPCs proliferation and ECM synthesis. **(A)** DNPCs proliferation detected by CCK-8 assay; **(B)** DNPCs apoptosis detected by flow cytometry; **(C)** expressions of ECM synthetic genes Aggrecan and Col II and ECM degrading genes MMP3 and ADAMTS5 detected by Western blot. Cell experiment was repeated three times. Data were presented as mean±standard error. Comparisons among groups were analyzed using one-way ANOVA, followed by Tukey’s multiple comparisons test. ^*^p<0.05, ^**^p<0.01.

To explore whether hBMSC-EVs affect DNPCs proliferation and apoptosis and ECM synthesis by transporting miR-129-5p, DNPCs were incubated with EVs in different groups for 24h. The results showed that DNPCs proliferation was reduced, apoptosis was increased, and ECM synthesis was decreased after silencing miR-129-5p in EVs (all *p*<0.05, Figures 4A–C). These results elicited that silencing miR-129-5p in EVs inverted the promoting effect of EVs on DNPCs proliferation and ECM synthesis, which further clarified that EVs promoted DNPCs proliferation and ECM synthesis by carrying miR-129-5p into DNPCs.

### miR-129-5p Targeted SOX4

**Figure 5:**
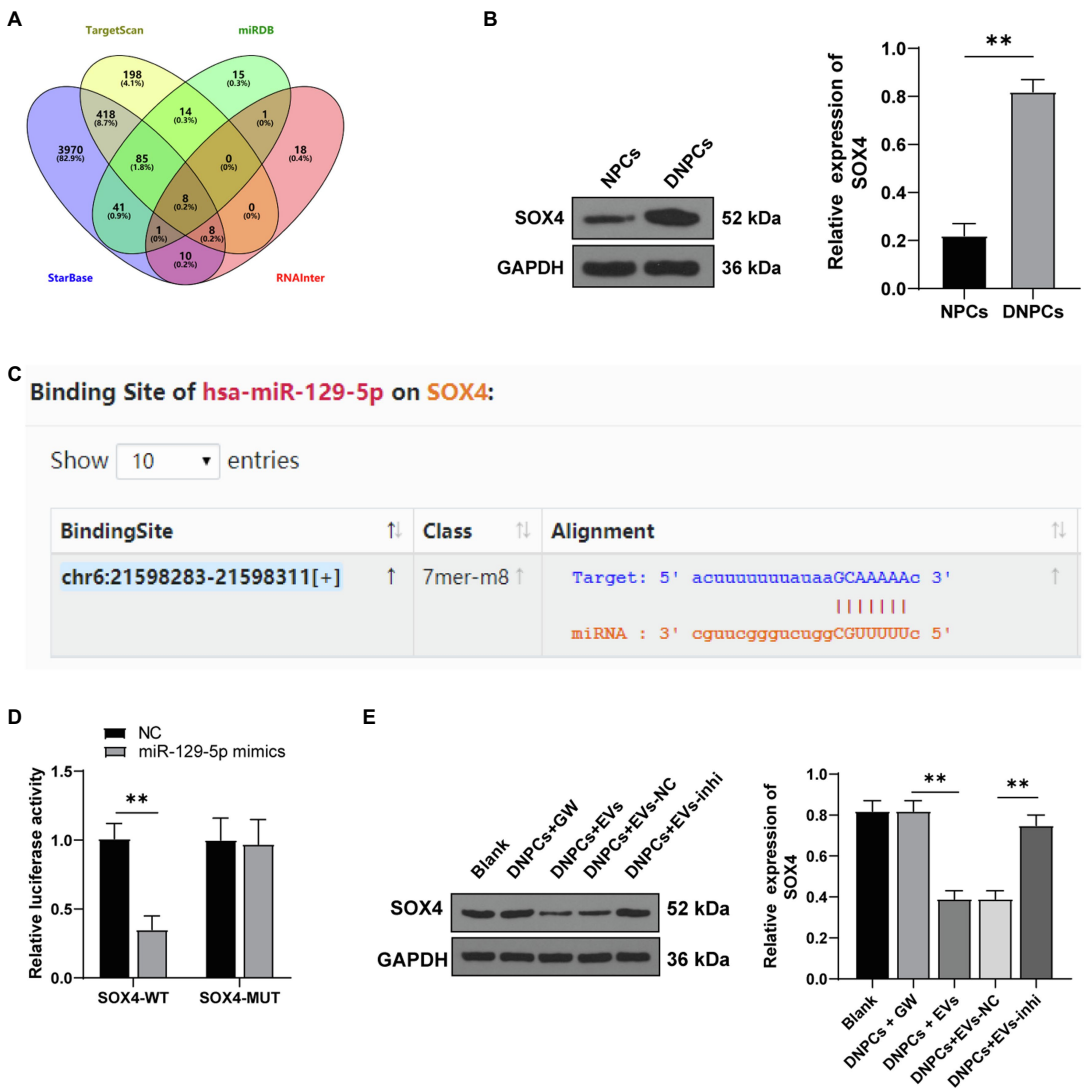
miR-129-5p targeted SRY-box transcription factor 4 (SOX4). **(A)** Eight downstream target genes of miR-129-5p screened through the StarBase, TargetScan, RNAInter, and miRDB databases; **(B)** SOX4 expression in NPCs and DNPCs detected by Western blot; **(C)** binding sites of miR-129-5p and SOX4 predicted on the StarBase database; **(D)** target relationship between miR-129-5p and SOX4 verified by dual-luciferase assay; **(E)** SOX4 expression in DNPCs detected by Western blot. Cell experiment was repeated three times. Data were presented as mean±standard error. Data in panels **(B,D)** were analyzed using independent *t* test, while data in panel **(E)** were analyzed using one-way ANOVA, followed by Tukey’s multiple comparisons test. ^**^p<0.01.

To investigate the downstream regulatory mechanism of EVs in promoting DNPCs proliferation and ECM synthesis *via* miR-129-5p, eight downstream target genes of miR-129-5p were identified by searching on the StarBase, TargetScan, RNAInter, and miRDB database (Figure 5A). Among the eight downstream target genes, SOX4 was reported to be upregulated in degenerated NP tissues relative to healthy nucleus pulposus tissues and have the ability to promote NPC apoptosis and inhibit ECM synthesis (Sun et al., 2019). Western blot showed that SOX4 expression in DNPCs was higher than that in NPCs (*p*<0.01, Figure 5B). Moreover, the target relationship between miR-129-5p and SOX4 3′UTR sequence was predicted on the StarBase (Figure 5C), and dual-luciferase assay verified the target relationship between miR-129-5p and SOX4 (*p*<0.01, Figure 5D). Besides, Western blot showed that SOX4 expression was decreased in DNPCs after EV treatment and increased after silencing miR-129-5p (all *p*<0.01, Figure 5E). Briefly, miR-129-5p targeted SOX4.

### SOX4 Overexpression Partially Inverted the Promoting Effect of EVs on DNPCs Proliferation and ECM Synthesis

**Figure 6:**
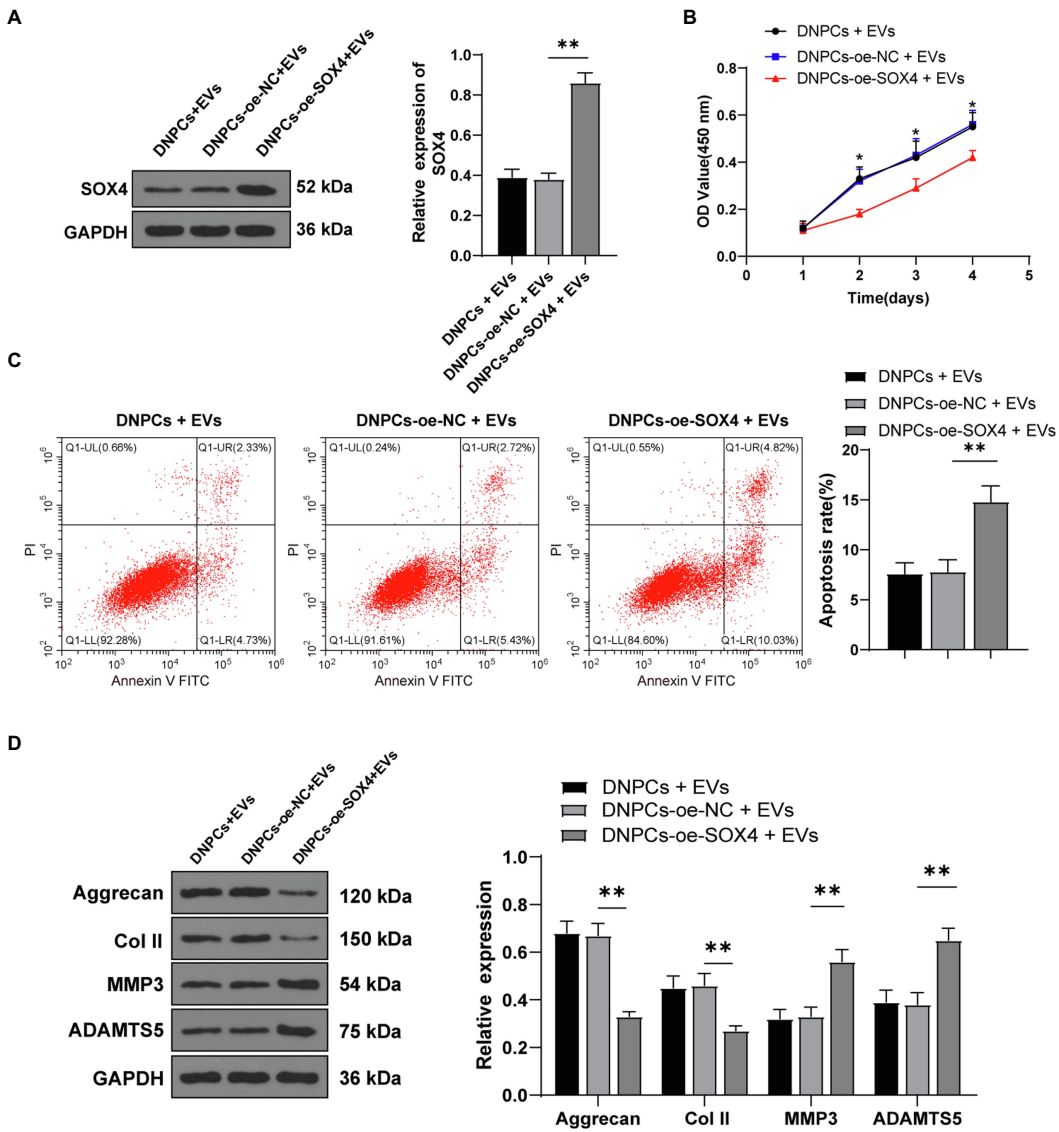
SRY-box transcription factor 4 overexpression partially inverted the promoting effect of EVs on DNPCs proliferation and ECM synthesis. **(A)** SOX4 expression in DNPCs detected by Western blot; **(B)** DNPCs proliferation detected by CCK-8 assay; **(C)** DNPCs apoptosis detected by flow cytometry; **(D)** expressions of ECM synthetic genes Aggrecan and Col II and ECM degrading genes MMP3 and ADAMTS5 detected by Western blot. Cell experiment was repeated three times. Data were presented as mean±standard error. Comparisons among groups were analyzed using one-way ANOVA, followed by Tukey’s multiple comparisons test. ^*^p<0.05, ^**^p<0.01.

Degenerated nucleus pulposus cells were transfected with SOX4 overexpression plasmid along with EV treatment. Western blot showed that SOX4 expression in the DNPCs-oe-SOX4+EVs group was increased compared with that in the DNPCs-oe-NC+EVs group (*p*<0.01, Figure 6A), indicating that SOX4 was successfully overexpressed. The promoting effect of EVs on DNPCs proliferation and ECM synthesis was inverted after overexpressing SOX4 (all *p*<0.05, Figures 6B–D). These results demonstrated that hBMSC-EVs downregulated SOX4 and thus promoted DNPCs proliferation and ECM synthesis and inhibited DNPCs apoptosis by carrying miR-129-5p into DNPCs.

### EVs Inhibited the Activation of Wnt/β-Catenin Pathway *via* the miR-129-5p/SOX4 Axis

**Figure 7:**
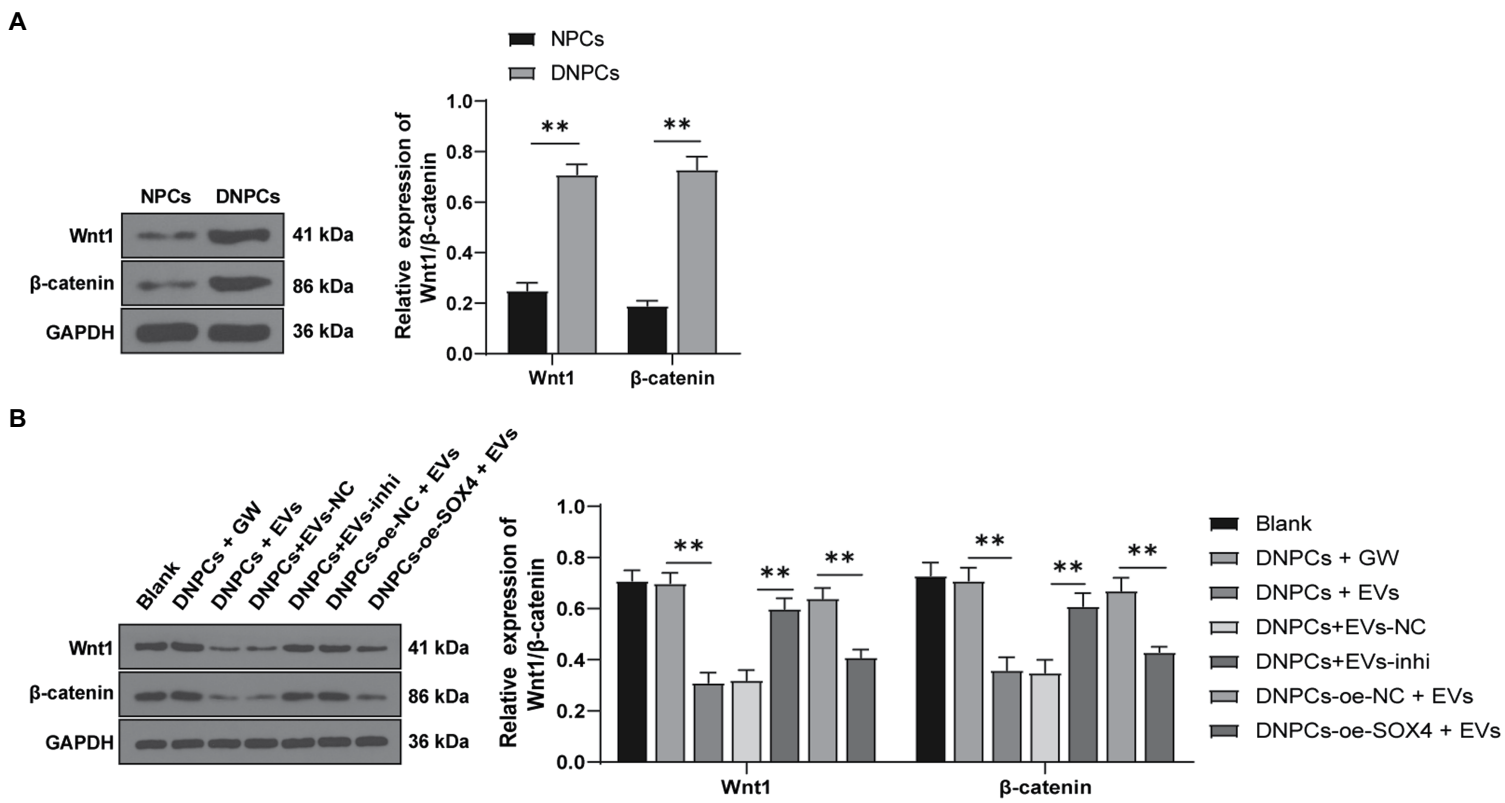
Extracellular vesicles inhibited the activation of Wnt/β-catenin pathway *via* the miR-129-5p/SOX4 axis. **(A)** Expressions of Wnt1 and β-catenin in NPCs and DNPCs detected by Western blot; **(B)** expressions of Wnt1 and β-catenin in DNPCs in different treatment groups. Cell experiment was repeated three times. Data were presented as mean±standard error. Data in panel **(A)** were analyzed using independent *t* test and data in panel **(B)** were analyzed using one-way ANOVA, followed by Tukey’s multiple comparisons test. ^**^p<0.01.

Wnt/β-catenin pathway has been reported to participate in IDD progression, and the activation of the Wnt/β-catenin pathway promotes NPC senescence, apoptosis, and ECM degradation (Chen et al., 2017; Zhan et al., 2019). To explore whether EVs regulate DNPCs proliferation, apoptosis, and ECM synthesis by activating the Wnt/β-catenin pathway *via* the miR-129-5p/SOX4 axis, expressions of Wnt1 and β-catenin in NPCs and DNPCs were detected. The result showed that expressions of Wnt1 and β-catenin in DNPCs were higher than those in NPCs (*p*<0.01, Figure 7A). Expressions of Wnt1 and β-catenin were decreased in EVs-treated DNPCs and increased after silencing miR-129-5p in EVs (all *p*<0.01, Figure 7B). These results exhibited that hBMSC-EVs inactivated the Wnt/β-catenin pathway through the miR-129-5p/SOX4 axis.

## Discussion

Intervertebral disk degeneration remains a chief cause of morbidity and disability and medical costs around the world (Kos et al., 2019). MSC-EVs are regarded as a novel therapeutic approach for IDD (Liao et al., 2021). The present study elicited that hBMSC-EVs promoted DNPCs proliferation and ECM synthesis by carrying miR-129-5p into DNPCs to target SOX4 and inhibit the activation of the Wnt/β-catenin axis.

After identification of hBMSCs and EVs, DNPCs were induced by TNF-α, and Dil-labeled EVs were cocultured with DNPCs. Our result showed that EVs were internalized by DNPCs after 24-h incubation. MSC-EVs are reported to inhibit NPC apoptosis, promote proliferation, and participate in matrix organization and distribution of ECM components (Rilla et al., 2019; Piazza et al., 2020). Our results found that the proliferative ability of EVs-treated DNPCs was enhanced, while DNPCs apoptosis was decreased. Col II is a main component of gelatinous tissues of nucleus pulposus (Chen et al., 2016). Destruction of Col II and Aggrecan is an important feature of IDD (Yang et al., 2020). MMP3 plays a central role in IDD and ADAMTS5 is the primary aggrecanase mediating IDD (Ngo et al., 2017; Zhao et al., 2020). Our results showed that expressions of Col II and Aggrecan were increased, while expressions of MMP3 and ADAMTS5 were decreased in DNPCs after EV treatment. MSC-EVs have been identified to modulate NPC proliferation and apoptosis (Cheng et al., 2018). EVs carrying miRNAs impact ECM degradation in osteoarthritis (Withrow et al., 2016). Consistently, our results demonstrated that hBMSC-EVs inhibited apoptosis and promoted proliferation of DNPCs and ECM synthesis.

miR-129-5p was reported to be involved in IDD progression and was differentially expressed in NPCs of IDD patients (Yang and Sun, 2019). Our result showed that miR-129-5p expression was decreased in DNPCs and elevated after EV treatment. Additionally, miR-129-5p expression in hBMSC-EVs was higher than that in GW-4869-treated hBMSC supernatant. To confirm whether hBMSC-EVs affect DNPCs proliferation and apoptosis and ECM synthesis *via* the delivery of miR-129-5p, EVs were treated with RNase and lysis buffer SDS. The results showed that miR-129-5p expression in EVs showed no significant difference after RNase treatment but was decreased in EVs treated with RNase and lysis buffer SDS, suggesting miR-129-5p was encapsulated by EVs. Subsequently, EVs were isolated from hBMSCs transfected with miR-129-5p inhibitor. The result showed that miR-129-5p expression was decreased significantly in transfected hBMSCs, isolated EVs, and DNPCs treated with EVs-inhi. Human synovial MSCs-derived exosomes relieved IL-1β-induced osteoarthritis by carrying miR-129-5p into chondrocytes (Qiu et al., 2021). Similarly, our results showed that hBMSC-EVs carried miR-129-5p into DNPCs. Furthermore, miR-129-5p knockdown promotes NPC apoptosis and causes collagen I synthesis (Chen et al., 2018; Li et al., 2020). Our results showed that EVs-treated DNPCs proliferation was decreased, and apoptosis and ECM synthesis decreased after silencing miR-129-5p in EVs. Zhao et al. have reported that miR-129-5p regulates cell apoptosis in nucleus pulposus in IDD (He et al., 2015). miR-129-5p serves as a negative regulator of collagen I in systemic sclerosis (Nakashima et al., 2012). Collectively, EVs promoted DNPCs proliferation and ECM synthesis by carrying miR-129-5p into DNPCs. Similar to miR-129-5p, miR-129-3p enhances the viability and restrains the apoptosis of chondrocytes in osteoarthritis (Chen et al., 2020). However, the involvement of miR-129-3p in IDD has not been reported. In addition, miR-129-3p and miR-129-5p exert similar effects in diseases *via* different mechanisms. For example, in the amelioration of diabetes, miR-129-3p decreases the apoptosis and recruitment of neutrophils by regulating the translation of Casp6 and Ccr2 (Umehara et al., 2019), whereas miR-129-5p inhibits ECM degradation by inhibiting the expression of Sp1-mediated MMP9 (Wang et al., 2018a).

To explore the downstream mechanism of EVs in DNPCs proliferation and ECM synthesis *via* miR-129-5p, downstream target genes of miR-129-5p were investigated. Among the screening results, SOX4 was reported to contribute to ECM and IDD progression (Zhang et al., 2021). Our results showed that SOX4 expression was increased in DNPCs. Target relationship between miR-129-5p and SOX4 was verified by dual-luciferase assay. Furthermore, SOX4 expression was decreased in EVs-treated DNPCs and increased after silencing miR-129-5p in EVs. Taken together, miR-129-5p targeted SOX4. Moreover, SOX4 overexpression plasmids were transfected into EVs-treated DNPCs to explore its effect. Upon SOX4 overexpression, the promoting effects of EVs on DNPCs proliferation and ECM synthesis were averted. According to Sun et al. (2019) SOX4 downregulation attenuates TNF-α-induced NPC apoptosis and balances anabolism and catabolism of ECM. SOX4 overexpression reverses the effect of circITCH depletion on enhancing NPC proliferation and expressions of Aggrecan and Col II (Zhang et al., 2021). These results demonstrated that hBMSC-EVs promoted DNPCs proliferation and ECM synthesis by carrying miR-129-5p into DNPCs to target SOX4.

The Wnt/β-catenin pathway is associated with NPC apoptosis and ECM degradation (Zhan et al., 2019). Our results showed that expressions of Wnt1 and β-catenin were increased in DNPCs. Adipose MSCs-derived exosomes promote cell proliferation and inhibit apoptosis *via* the Wnt/β-catenin pathway in cutaneous wound healing (Ma et al., 2019). Our results showed that expressions of Wn1 and β-catenin were decreased in EVs-treated DNPCs and increased after silencing miR-129-5p in EVs. miR-129-5p suppresses the Wnt/β-catenin pathway in osteoblasts (Yin et al., 2020). circITCH upregulates SOX4 to activate the Wnt/β-catenin pathway in IDD (Zhang et al., 2021). Conjointly, hBMSC-EVs inhibited the activation of Wnt/β-catenin pathway *via* the miR-129-5p/SOX4 axis.

In conclusion, this study elicited that hBMSC-EVs promoted DNPCs proliferation and ECM synthesis by carrying miR-129-5p into DNPCs to target SOX4 and inhibit the activation of the Wnt/β-catenin pathway. However, this study has its limitations. Firstly, the effect of hBMSC-EVs on DNPCs proliferation and ECM synthesis was verified through *in vitro* experiment only. Animal experiment and clinical validation are absent. Secondly, the age factor was not considered. The occurrence of IDD is closely associated with age, and age could affect the functioning of NPCs (Lehmann et al., 2014; Tang et al., 2019; Wang et al., 2020). In addition, the secretion of miRNAs in BMSCs-derived exosomes changes with aging (Davis et al., 2017; Xun et al., 2021). Hence, animal experiment and clinical trials are to be performed and human NPCs and hBMSCs from patients of different ages are to be used in future studies. Besides, upstream long non-coding RNAs of miR-129-5p or other miRNAs in EVs and other downstream target genes and pathways related to miR-129-5p should be investigated in future studies.

## Data Availability Statement

The raw data supporting the conclusions of this article will be made available by the authors, without undue reservation.

## Author Contributions

HW contributed to the study concepts, study design, and definition of intellectual content. FL and WB contributed to the literature research. HW and FL contributed to the manuscript preparation. JZ contributed to the manuscript editing and review. WB and JZ contributed to the experimental studies and data acquisition. HW and GZ contributed to the data analysis and statistical analysis. All authors contributed to the article and approved the submitted version.

## Funding

This study was supported by grant from the Shaanxi Provincial Key Research and Development Program (No. 2020SF-085).

## Conflict of Interest

The authors declare that the research was conducted in the absence of any commercial or financial relationships that could be construed as a potential conflict of interest.

## Publisher’s Note

All claims expressed in this article are solely those of the authors and do not necessarily represent those of their affiliated organizations, or those of the publisher, the editors and the reviewers. Any product that may be evaluated in this article, or claim that may be made by its manufacturer, is not guaranteed or endorsed by the publisher.

## Footnotes

^1^ http://starbase.sysu.edu.cn/

^2^ http://www.targetscan.org/vert_71/

^3^ http://www.rna-society.org/rnainter/

^4^ http://www.mirdb.org/
